# Supplementary material for: A regularized functional regression model enabling transcriptome-wide dosage-dependent association study of cancer drug response
Source: PLoS Comput Biol. 2021 Jan 25;17(1):e1008066. doi: 10.1371/journal.pcbi.1008066 (PMC7920352; doi:10.1371/journal.pcbi.1008066)
Supplement: S1 Text — Simulated responses have been generated to examine the accuracy of the employed method in detecting the genes that are truly associated to drug response. Three screening thresholds, three active gene sets and two covariance structure scenarios for the repeated measurements simulation have been considered. This text includes all the details of the simulation study that we conducted. (PDF) [file pcbi.1008066.s001.pdf]

Supporting information S1 Text:

Accurate detection of drug associated genes from simulated responses

Evanthia Koukouli[1*], Dennis Wang [2,3], Frank Dondelinger[4], Juhyun Park[1]

1 Department of Mathematics and Statistics, Fylde College, Lancaster University, Bailrigg, Lancaster, UK

2 Sheffield Institute for Translational Neuroscience, University of Sheffield, Sheffield, UK

3 Department of Computer Science, University of Sheffield, Sheffield, UK

4 Centre for Health Informatics and Statistics, Lancaster Medical School, Lancaster University, Bailrigg, Lancaster, UK

*e.koukouli@lancaster.ac.uk (EK)

Monte Carlo simulations have been conducted to examine the ability of our model to detect the genes that are truly associated with the drug response. Due to the computational burden associated with a simulation of the same scale as the dataset, we conducted a simulation using a smaller simulated dataset. In each iteration, a random sample without replacement of size $n_s = [p_1 n]$ experimental units and $G_s = [p_1 G]$ genes ($p_1 \in [0, 1]$) is extracted from the original data set. Responses over different dosage levels are then generated based on a subset of G_s, $G_s^A = [p_2 G_s]$, and some low-dimensional covariates from the original GDSC data (drug type and cancer cell line histology):

$$y_{ij}^s = \sum_{k=0}^{p} z_{ik}\beta_k(d_{ij}) + \sum_{g=1}^{G_s^A} x_{ig}\gamma_g(d_{ij}) + \varepsilon_{ij} \quad \text{with} \quad i = 1, \ldots, n_s, j = 1, \ldots, n_i \tag{1}$$

where $\{z_{ik} : i = 1, \ldots, n_s, k = 0, \ldots, p\}$ are the dose-invariant low-dimensional covariates and $\{x_{ig} : i = 1, \ldots, n_s, g = 1, \ldots, G_s^A\}$ represent the genetic information truly associated with the drug-response (active genes). Coefficient functions $\{\beta_k(d) : k = 0, \ldots, p\}$ and $\{\gamma_g(d) : g = 1, \ldots, G_s^A\}$ are, then defined as follows:

$$\beta_0(d) = 15 + 20\sin\left(\frac{30\pi d}{15}\right), \; \beta_1(d) = 15 + 20\cos\left(\frac{30\pi d}{15}\right)$$

$$\beta_2(d) = 2 - 3\cos\left(\frac{\pi(30d-25)}{15}\right),\ \beta_3(d) = 2 - 3\sin\left(\frac{\pi(30d-25)}{15}\right)$$

$$\beta_4(d) = 6 - 0.2(30d)^2,\ \beta_5(d) = -4\frac{(20-30d)^3}{2000},\ \beta_6(d) = \sin(0.3\pi) + 0.4d^2$$

$$\beta_7(d) = 3 + \frac{(1-d)^2}{5},\ \beta_8(d) = 0.1\exp\left(-\frac{(1-d)^2}{2}\right),\ \beta_9(d) = 0.1\exp\left(\frac{(1-d)^2}{2}\right)$$

$$\beta_{10}(d) = \sin\left(\frac{\pi d}{15}\right) + \cos\left(\frac{\pi d}{15}\right),\ \beta_{11}(d) = 1 + 52d + 4d^2,\ \beta_{12}(d) = d^3 - 1$$

$$\gamma_g(d) = (d+1)^{\frac{r_g}{100}} \text{ where } r_g \sim Unif(1, G_s^A),\ \forall g \in G_s^A.$$

For any $g \in G_s^I,\ G_s^I = G_s \setminus G_s^A$ we assume $\gamma_g(d) = 0$. We, also, consider a rational quadratic covariance structure $\text{Cov}(\varepsilon_i(d), \varepsilon_i(s)) = \sigma^2 \frac{1}{1+(\frac{|d-s|}{r})^2}$ and a dose-varying variance $V(d) = \sigma^2 \frac{\sin^2 d}{1+(\frac{\cos d}{10})^2} + \sin\frac{d}{5}$ where $\sigma^2 = 0.001$.

We set $p_1 = 0.05$ resulting in an analytic sample of 190 experimental units and 886 genes. We consider 3 different scenarios for the number of active genes: $p_2 \in \{0.1, 0.05\}$ and $G_s^A = 1$. We repeatedly sampled experimental units and genes, and generated responses over five or nine dosage levels as described above. The performance of the employed methodology has been assessed based on 1000 simulations using different screening thresholds $\tau_n(\nu)$ and estimated covariance structure scenarios. Cubic B-splines and knots placed at the median of the observed data values have been used for estimating the coefficient functions.

Our method has utility for drug development if it robustly identifies genes truly associated with drug response. Fig 1 illustrates gene selection results from simulated drug responses that were generated by varying numbers of gene predictors. Our simulation shows that the sparser the vector of high-dimensional predictors, the better the performance of the algorithm (a result which makes sense since in both stages of the

algorithm the sparsity assumption is present and needs to be satisfied). As for the screening threshold selection, we observed that higher thresholds demonstrate better algorithm performance. The biggest differences were observed especially when the signal to noise ratio increases where the automatic screening threshold selection (Greedy-INIS algorithm [1]) seems not to be efficient enough. Small differences were observed when the covariance structure is not successfully estimated, however, bigger differences are expected when it comes to prediction accuracy. There were a few cases where the employed algorithm failed to identify the truly associated genes; however, this happened less than 0.01% of the simulations.

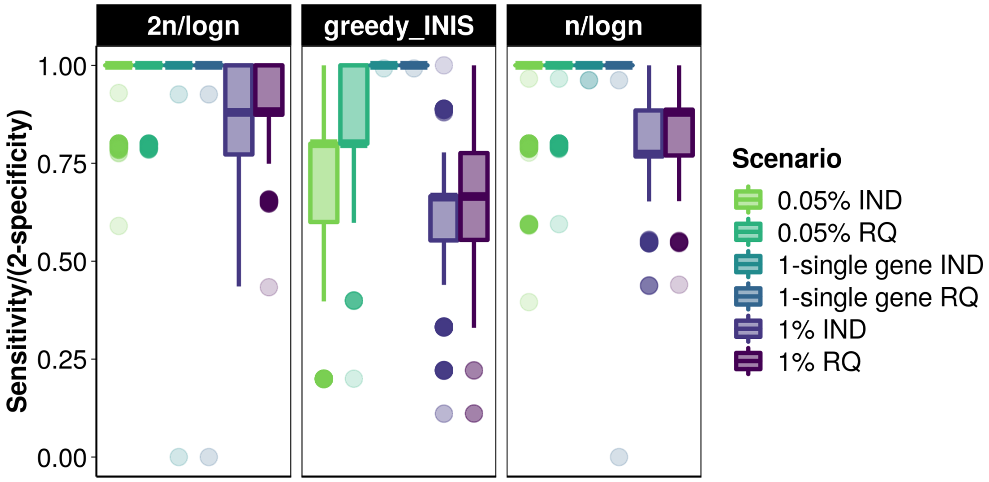

Fig 1. Pilot simulation study for optimal parameter tuning. Accuracy of detecting drug-gene associations using simulated data under different screening thresholds, covariance structure and G_s^A scenarios. Screening thresholds considered were: $\frac{n}{log(n)}$; $\frac{2n}{log(n)}$, and; a threshold proposed by applying the automated Greedy-INIS algorithm [1]—here, n is the number of experimental units in the data. The covariance structure scenarios were independence (IND) and rational quadratic (RQ). The number of active genes scenarios were: either one single gene; 0.5% of the genes in the data, or; 1% of the genes in the data.

References

1. Fan J, Feng Y, Song R. Nonparametric independence screening in sparse ultra-high-dimensional additive models. Journal of the American Statistical Association. 2011;106(494):544–557.
