# Supplementary figures and images for: A regularized functional regression model enabling transcriptome-wide dosage-dependent association study of cancer drug response
Source: PLoS Comput Biol. 2021 Jan 25;17(1):e1008066. doi: 10.1371/journal.pcbi.1008066 (PMC7920352; doi:10.1371/journal.pcbi.1008066)

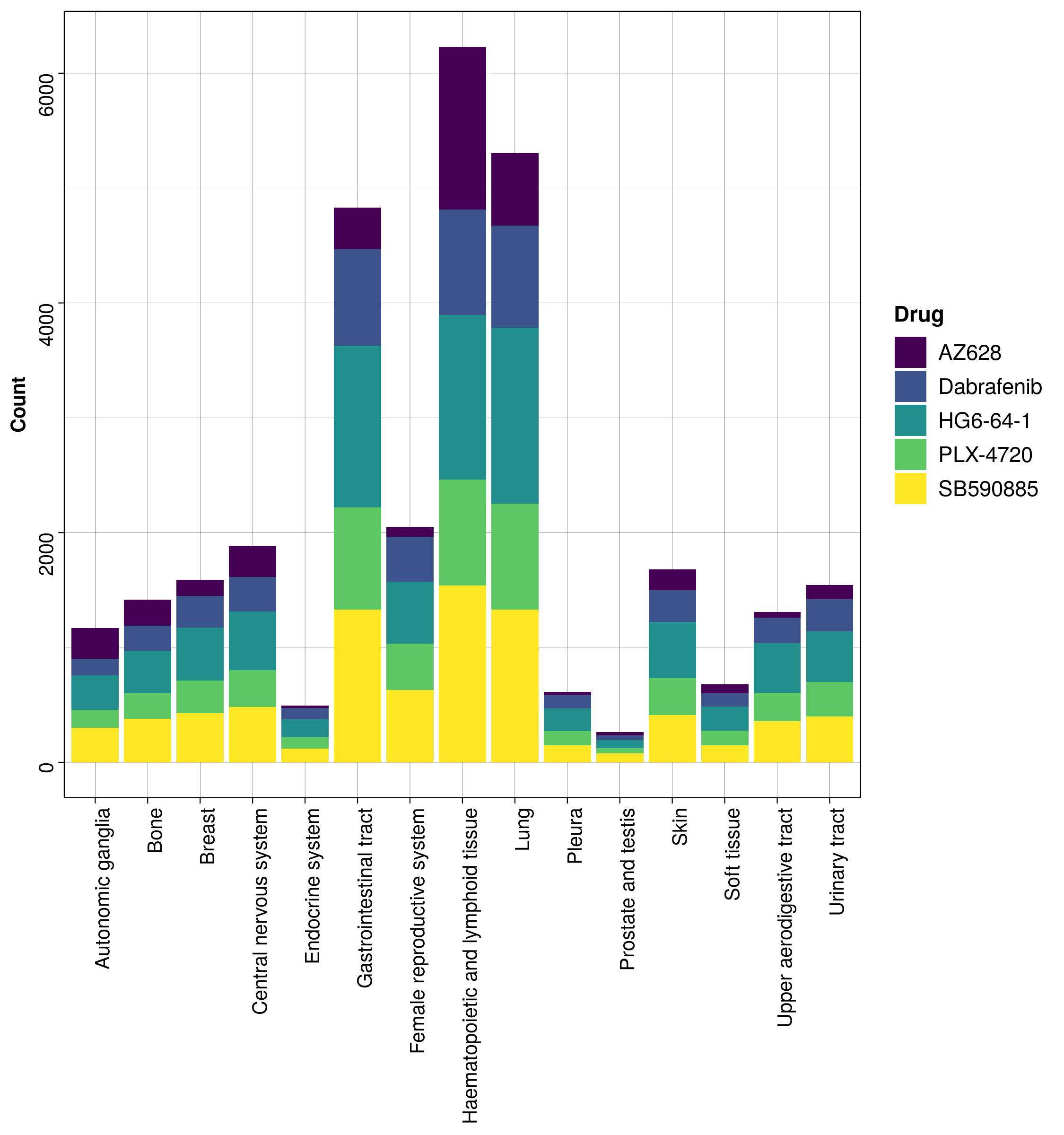

Supplement: S1 Fig — Overall, similar proportion of cell lines have been treated with all of the compounds examined with smaller number of cell lines been treated with AZ628, Dabrafenib and PLX-4720. Larger number of cell lines in the data set were originated from the lungs, the gastrointestinal tract and the haematopoietic and lymphoid tissues. (TIF) [file pcbi.1008066.s002.tif]

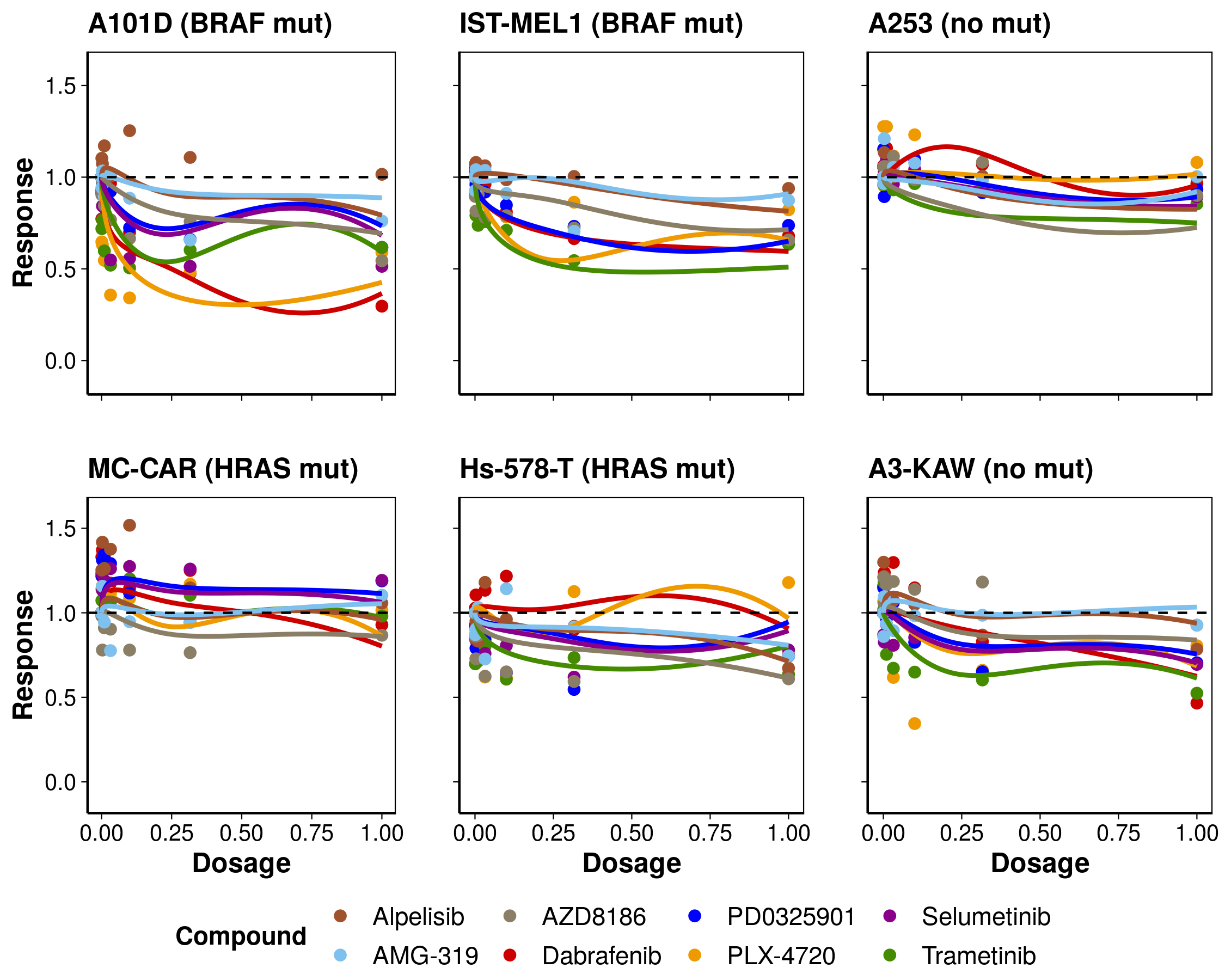

Supplement: S2 Fig — Observed responses (points) and estimated mean trajectory (lines) of cell concentration for cancer cell lines with and without BRAF and HRAS mutations after treatment with the eight anticancer compounds examined using data from GDSC2. (TIF) [file pcbi.1008066.s003.tif]

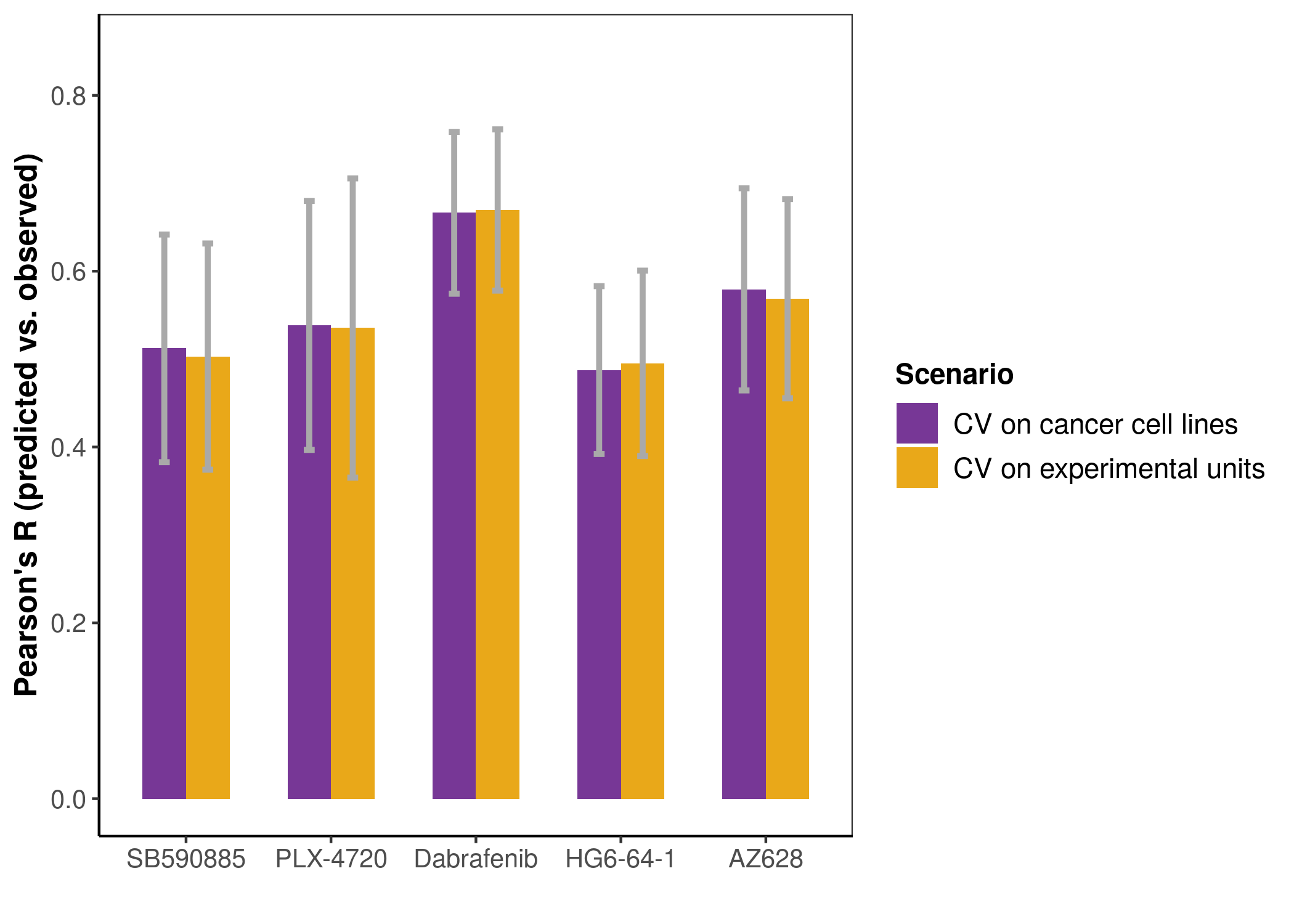

Supplement: S3 Fig — Pearson correlation was estimated across observed and predicted AUC values. AUC values have been computed by calculating the area under the coefficient function curve (both observed and predicted). Training and test sets have been considered based on either the experimental units or on cancer cell lines only. (TIF) [file pcbi.1008066.s004.tif]

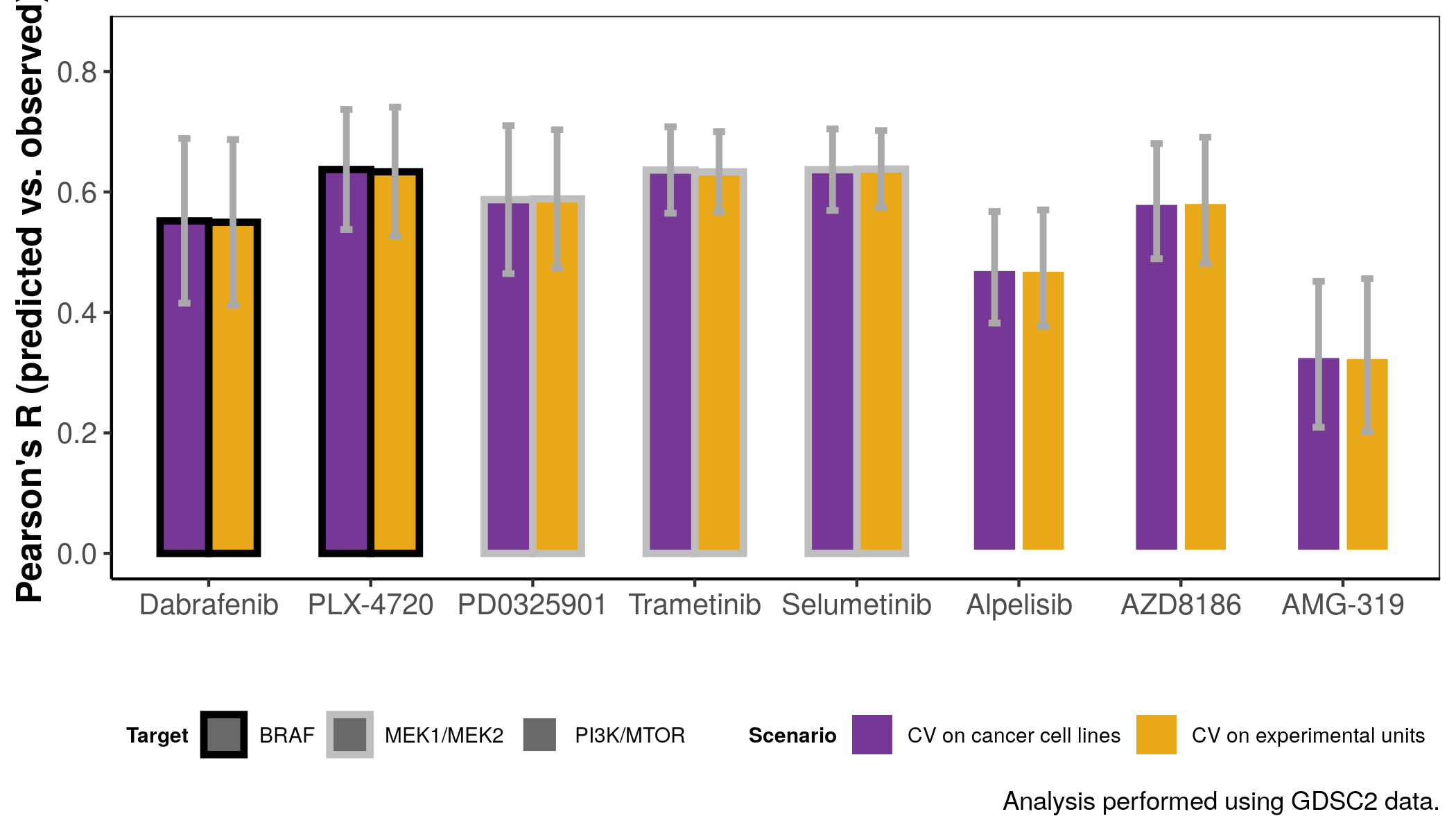

Supplement: S4 Fig — Pearson correlation across observed and predicted AUC values. AUC values have been computed by calculating the area under the coefficient function curve (both observed and predicted) using the GDSC2 data. Training and test sets have been considered based on either the experimental units or on cancer cell lines only. (TIF) [file pcbi.1008066.s005.tif]
